# The Glutamate-gated Chloride Channel Facilitates Sleep by Enhancing the Excitability of Two Pairs of Neurons in the Ventral Nerve Cord of *Drosophila*

**DOI:** 10.1007/s12264-025-01397-1

**Published:** 2025-04-30

**Authors:** Yaqian Fan, Yao Tian, Junhai Han

**Affiliations:** 1 School of Life Science and Technology, The Key Laboratory of Developmental Genes and Human Disease, Southeast University, Nanjing, 210096 China; 2 Co-innovation Center of Neuroregeneration, Nantong University, Nantong, 226000 China

**Keywords:** Sleep, Neural circuit, Glutamate-gated chloride channel, *Drosophila*, Ventral nerve cord, Neural activity

## Abstract

Sleep, an essential and evolutionarily conserved behavior, is regulated by numerous neurotransmitter systems. In mammals, glutamate serves as the wake-promoting signaling agent, whereas in *Drosophila*, it functions as the sleep-promoting signal. However, the precise molecular and cellular mechanisms through which glutamate promotes sleep remain elusive. Our study reveals that disruption of glutamate signaling significantly diminishes nocturnal sleep, and a neural cell-specific knockdown of the glutamate-gated chloride channel (GluClα) markedly reduces nocturnal sleep. We identified two pairs of neurons in the ventral nerve cord (VNC) that receive glutamate signaling input, and the GluClα derived from these neurons is crucial for sleep promotion. Furthermore, we demonstrated that GluClα mediates the glutamate-gated inhibitory input to these VNC neurons, thereby promoting sleep. Our findings elucidate that GluClα enhances nocturnal sleep by mediating the glutamate-gated inhibitory input to two pairs of VNC neurons, providing insights into the mechanism of sleep promotion in *Drosophila*.

**Supplementary Information:**

The online version contains supplementary material available at 10.1007/s12264-025-01397-1.

## Introduction

Sleep, a fundamental and evolutionarily conserved behavior, is evident from worms to humans [1, 2]. *Drosophila*, which displays sleep-like behavior, serves as a valuable model system for genetic studies [3–5]. The regulation of sleep in *Drosophila* is controlled by both circadian and homeostatic mechanisms [6, 7], paralleling the situation in mammals. As such, it has been utilized to explore the molecular regulation and mechanism of sleep [6, 8–10]. A range of neurotransmitter systems [11–13], circuits [14, 15], and biological processes [9, 16, 17] that impact sleep have been identified.

Glutamate serves as the primary excitatory neurotransmitter in both mammals and *Drosophila*. While some studies posit that glutamate functions as a wake-promoting neurotransmitter in mammals, its role in *Drosophila* sleep remains less explored and conclusions drawn are often contradictory. For instance, increasing the activity of glutamatergic neurons in the adult brain results in a significant reduction in both daytime and nighttime sleep [18], suggesting that glutamate may act as a wake-active neurotransmitter. However, genetic knockdown of *Drosophila* glutamate receptor A (GluRA) has been found to increase daytime sleep and reduce nighttime sleep [19], while knockdown of *Drosophila* N-methyl-D-aspartate receptor 1 (NMDAR1) reduces both daytime and nighttime sleep [20]. These findings imply that glutamate may function as a sleep-promoting neurotransmitter. Glutamate can trigger excitatory impulses by activating glutamate receptors on the postsynaptic membrane of glutamatergic neurons [21] and may increase GABAergic inhibitory output across the circuit by activating glutamate receptors on the membrane of GABAergic neurons [22]. Furthermore, glutamate may also function as an inhibitory neurotransmitter in the fly central nervous system [23, 24]. Therefore, the comprehensive effect of glutamate on sleep regulation is contingent upon the specific location and the type of glutamate receptors.

In this study, we demonstrate that neural cell-specific knockdown of the vesicular glutamate transporter (vGluT) diminishes nocturnal sleep. We also reveal that depletion of Glu-gated chloride channel-α (GluClα) significantly reduces nocturnal sleep. We identified two pairs of ventral nerve cord (VNC) neurons that receive glutamate signaling input and found that GluClα depletion from these neurons decreased nighttime sleep. Finally, we showed that GluClα mediates the glutamate-gated inhibitory input to these VNC neurons and that increasing their activity reduces nighttime sleep. Our findings suggest that GluClα enhances nocturnal sleep by mediating the glutamate-gated inhibitory input to two pairs of VNC neurons, and thereby provide insights into the mechanism of sleep promotion in *Drosophila*.

## Materials and Methods

### Fly Stocks and Maintenance

*Drosophila* strains were crossed and maintained on standard yeast-agar medium under a 12:12 LD (light/dark) cycle at 25℃, with a relative humidity of 65%. *TH-D4-GAL4* flies were from Dr. Mark N. Wu (Johns Hopkins University, Baltimore, USA): *R6-GAL4* flies from Dr. Paul Taghert (Washington University, St. Louis, USA): *vGluT-Trojan-GAL80* flies from Dr. Zhihua Liu (Hubei University, Hubei, China); *Otd-FLP,tubP>stoP > GAL80*, *34F06-GAL4*, and *34F06-LexA* flies from Dr. Fang Guo (Zhejiang University, Zhejiang, China); *GluClα-KO-LexA* and *CNMa-GAL4* flies from Dr. Yi Rao (Capital Medical University, Beijing, China); *UAS-Dicer* from Dr. Pengyu Gu (Sir Run Run Shaw Hospital, Zhejiang, China); *Tsh-GAL80* flies from Dr. Wei Xie (Southeast University, Nanjing, China); *LexAop-GFP*^*11*^*;UAS-GFP*^*1-10*^ flies from Dr. Chuan Zhou (Institute of Zoology, Beijing, China); and *vGluT-LexA, Lk-GAL4* flies from Dr. Yufeng Pan (Southeast University, Nanjing, China). The wild-type flies used in this work were *w*^*1118*^. The sources of *Drosophila* strains are listed in Table 3.

### Behavioral Assays

All behavior analyses were conducted on male flies. Fresh males were collected and raised together on a standard yeast-agar medium under a 12:12 LD cycle at 25℃. After 3–5 days, the collected flies were placed in a transparent glass tube, with sleeping food (1.5% agar and 5% sucrose) at one end and a cotton ball at the other end to limit the activity space of the fly. The tube containing the fly was placed in a fruit fly activity detector, which was placed in a 25℃, 12 LD incubator. The data were analyzed using the Sleep and Circadian Analysis MatLab Program [25].

### Two-photon Calcium Imaging

Fruit flies aged 3-5 days were collected and the brain and VNC were dissected in HL3 buffer (in mmol/L: 20.0 MgCl_2_, 5 KCl, 70 NaCl, 10.0 NaHCO_3_, 1.5 CaCl_2_, 5 trehalose, 115 sucrose, and 5.0 HEPES, at pH 7.2) [26]. An isolated brain and VNC were placed on a glass slide (Citotest, China) with a drop of HL3 buffer and imaged under a Zeiss LSM 980 confocal microscope. Under the microscope, the target neurons were located, laser scanning was paused, the neurons were allowed to adapt for 2 min, and then the scanning and imaging were resumed. Once the baseline was stable, a micropipette was used to add glutamate solution (pH 7.2) near the cell body. All the imaging data were analyzed using the ROI processing module in Zeiss software. The ΔF/F0 was calculated, where F0 represents the baseline average fluorescence and ΔF is F *minus* F0.

### Immunostaining and Imaging

Files were anesthetized with CO_2_, and each brain and VNC was dissected in ice-cold phosphate-buffered saline (1×PBS) and fixed with 4% paraformaldehyde (PFA) for 20 min at room temperature. Fixed files were washed 3 times in PBST (0.3% Triton X-100) and then blocked with PBS containing 1% goat serum for 40 min. After blocking, the brain and VNC were incubated overnight at 4℃ in primary antibody, washed 3 times in 0.3% PBST, incubated for 2 h at room temperature in secondary antibody, and then washed in PBST. Finally, the sealing solution was applied and the tissues were mounted on a glass slide. The primary antibodies were: mouse anti-Bruchpilot (1:30, DSHB #nc82), anti-GFP polyclonal antibody, Alexa Fluor 488 (1:200, Invitrogen #A21311), mouse anti-DsRed (1:200, Santa Cruz Biotechnology #sc-390909), and mouse anti-GFP (1:100, Sigma-Aldrich #G6539). The Alexa Fluor secondary antibodies were: goat anti-rabbit 488 (1:200; Abcam #ab150081), goat anti-mouse 488 IgG (1:200; Abcam #ab150117), goat anti-rabbit 647 (1:200; Abcam #ab150083), and goat anti-mouse 647 (1:200; Abcam #ab150115). Fluorescence images were acquired using a Zeiss LSM 900 confocal microscope and processed in ZEN lite.

### Sleep Deprivation

3-5-day-old male fruit flies were placed in the TriKinetics *Drosophila* Activity Monitor System (DAM2; TriKinetics, USA), and a mechanical device was used to deprive them of sleep. The baseline sleep was recorded for one day at 25℃ and then they were mechanically sleep-deprived during ZT12-ZT24 on the second night, starting from ZT0. On the third day, they were placed in a DigiTherm CircKinetics incubator (Tritech Research, USA), and the sleep rebound within the next 12 or 24 h (ZT0-ZT12 or ZT0-ZT24) was recorded. Flies with <80% sleep loss on deprivation were excluded from the analysis.

### RNAi Screening

To validate the gene nomenclature of glutamate receptors in *Drosophila*, we reviewed the literature and categorized targets into three conserved classes (kainate, AMPA, and NMDA types) of cationic iGluRs, metabotropic glutamate receptors (mGluRs) and one chloride channel (GluClα), with associated RNAi lines listed in Table 1. Fruit flies were supplied by the Tsinghua Fly Center and Bloomington *Drosophila* Stock Center. After mating *nSyb-GAL4* with different *UAS-RNAi* flies, their offspring were collected, raised for 3–5 days, and then used for sleep experiments.

### Quantification and Statistical Analysis

Statistical analyses were applied with GraphPad Prism V8.0.2 software. Two methods of analysis were applied: one for normally-distributed data normal distribution and another for data analysis that did not conform to the normal distribution. Normally distributed data were analyzed using the parametric statistic, a one-way analysis of Dunnett’s multiple comparisons for the differences among the three genotypes. For the data with a non-normal distribution, the Kruskal-Wallis test by Dunn’s multiple comparisons was used to assess the difference among the three genotypes. Statistically significant differences were set as: *P < 0.05, **P < 0.01, ***P < 0.001; ns, no significant difference, ^†^*P > *0.05.

**Fig. 1:**
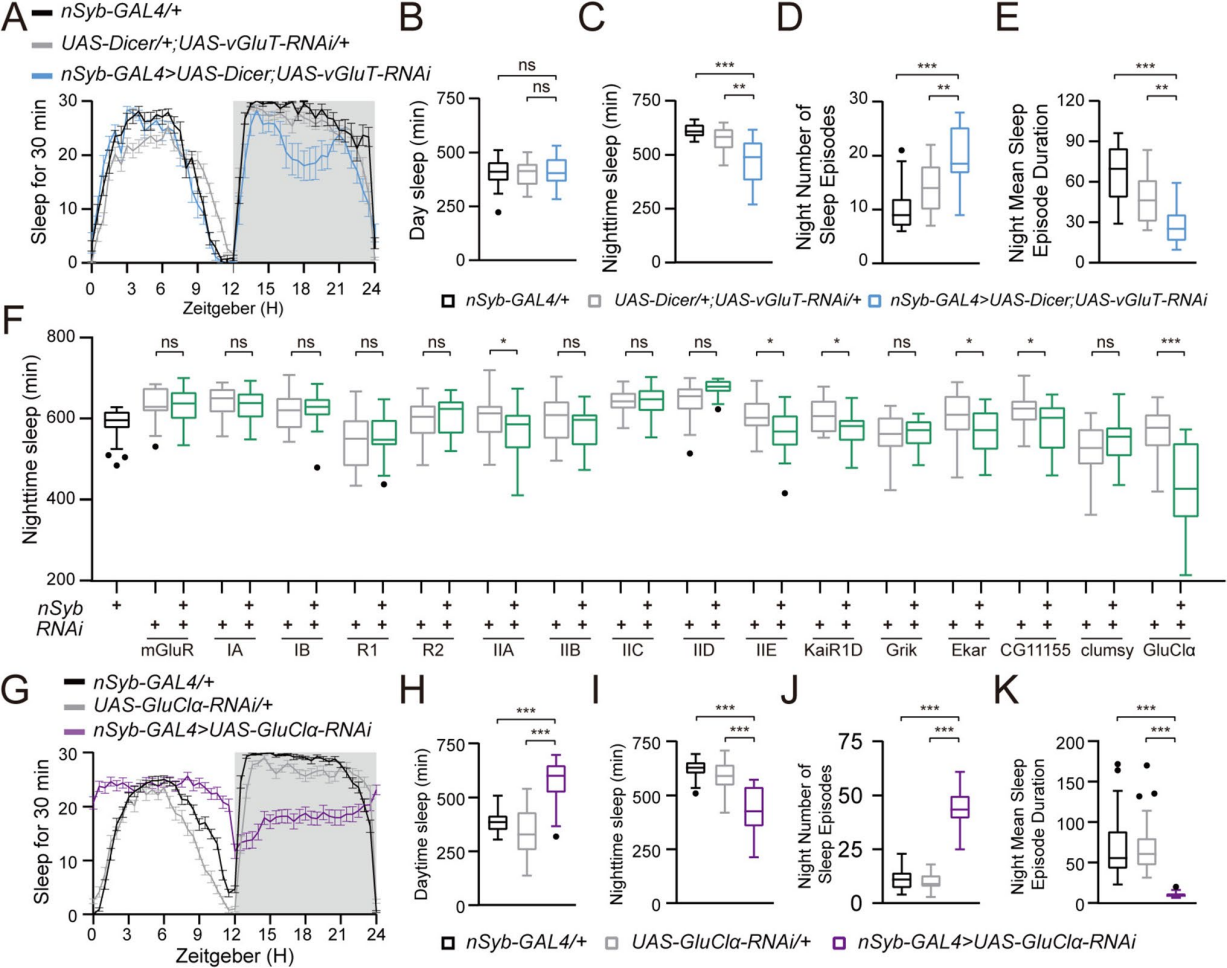
GluClα expression in neurons is essential for nighttime sleep. **A** Sleep traces of control *w;nSyb-GAL4/+* (black, *n =* 16), *w;UAS-Dicer/+;UAS-vGluT-RNAi/+* (light gray, *n = *27), *w;UAS-Dicer/+;nSyb-GAL4/UAS-vGluT-RNAi* (blue, *n = *14), plotted as a 30-min moving average. **B-E** Total day sleep **B**, total nighttime sleep **C**, number of night sleep episodes **D**, and mean night sleep episode duration **E** for each genotype. **F** Total nighttime sleep of flies of each genotype with GluR knockdown using a *nSyb-GAL4* driver and different *UAS-GluR-RNAi*s. A single copy of the GAL4 driver was used for each RNAi line. **G** Sleep traces of control *w;;nSyb-GAL4/+* (black, *n = *50), *w;UAS-GluClα-RNAi/+* (light gray, *n = *52), *w;UAS-GluClα-RNAi/+;nSyb-GAL4/+* (purple, *n = *55) plotted as a 30-min moving average. **H-K** Total day sleep **H**, total nighttime sleep **I**, number of night sleep episodes **J**, and mean night sleep episode duration **K** for each genotype. For **B**, **C**, **F**, **H**, **I**, one-way ANOVA with Dunnett’s post hoc, ns, *P > *0.05, ***P < 0.001; for **D**, **E**, **J**, **K**, Kruskal-Wallis followed by Dunn’s multiple comparison test, ns, *P > *0.05, ***P < 0.001.

## Results

### Depletion of GluClα Markedly Diminishes Nocturnal Sleep

To investigate the potential roles of glutamate signaling in *Drosophila* sleep regulation, we applied RNAi against the vGluT, a crucial molecule for loading glutamate into synaptic vesicles [27]. Knockdown of vGluT using pan-neuronal *nSyb-GAL4* significantly reduced nocturnal sleep (Fig. 1A–C). Furthermore, compared with the control flies, *nSyb-GAL4>UAS-vGluT-RNAi* flies exhibited more night sleep episodes with markedly shorter durations (Fig. 1D, E), reflecting that glutamate signaling plays critical roles in consolidating nocturnal sleep. These findings suggest that glutamate signaling enhances nocturnal sleep in *Drosophila*. To pinpoint the glutamate receptor that mediates this signaling, we screened 14 known ionotropic glutamate receptor (iGluR) subunits, encompassing three conserved classes (kainate, AMPA, and NMDA types) of cationic iGluRs, metabotropic glutamate receptors (mGluRs) and one chloride channel (GluClα) [28] (Table 1; Fig. 1F). Unexpectedly, neuronal knockdown of GluClα using the pan-neuronal *nSyb-GAL4* significantly reduced nocturnal sleep (Fig. 1G-I), increased nocturnal sleep episodes (Fig. 1J), and shortened nocturnal sleep durations (Fig. 1K). However, neuronal knockdown of other glutamate receptors had partial or no effects on nocturnal sleep (Fig. 1F).

**Fig. 2:**
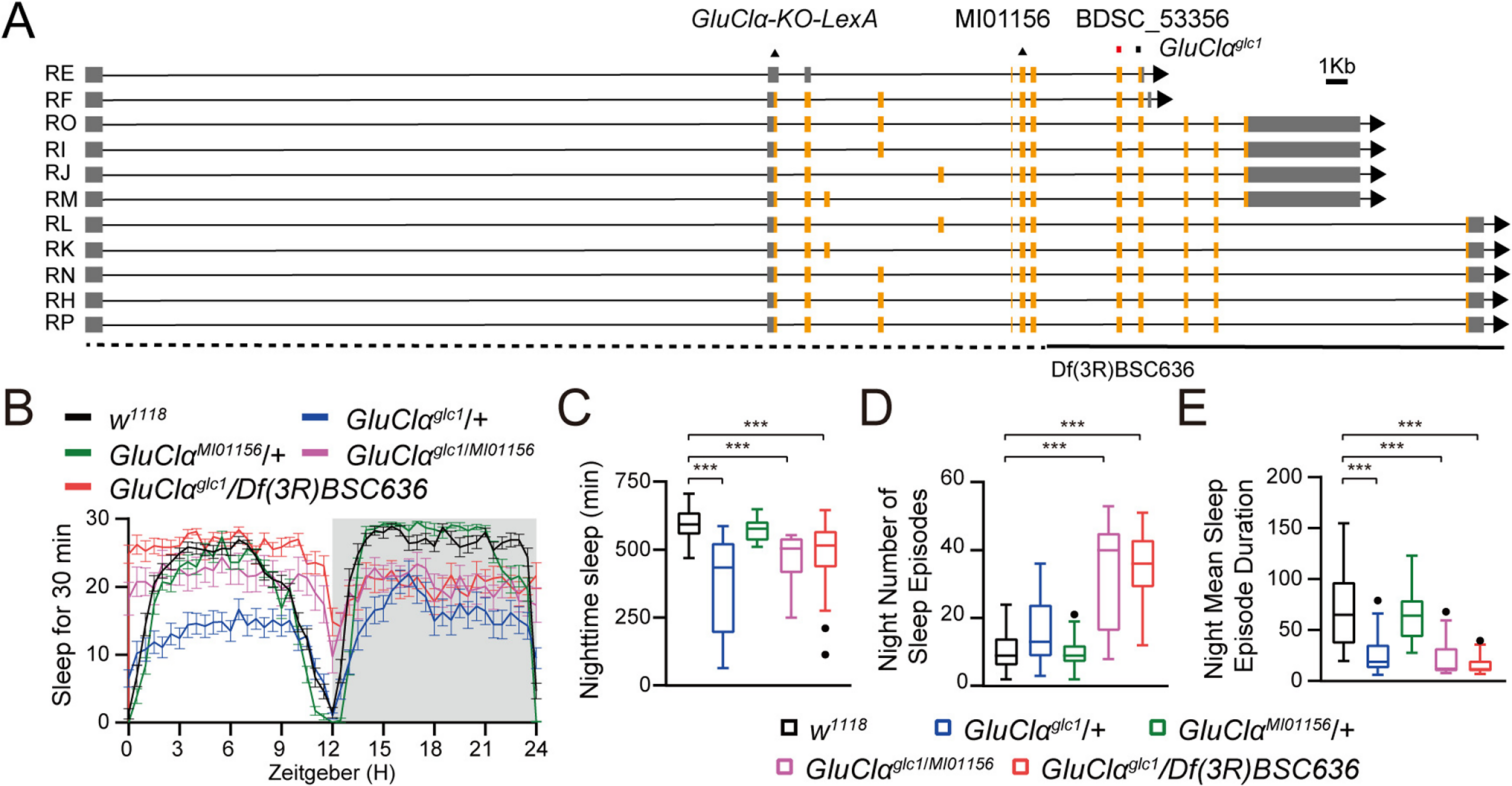
GluClα mutant flies exhibit reduced nighttime sleep. **A** Annotated transcription of the GluClα gene. Black triangle, the MI01156 insertion site; red square, the RNAi target site; black square, the point mutant site; dashed lines, the deleted regions of Df(3R)BSC636. The LexA insertion site is labeled above. **B** Sleep traces of control *w*^*1118*^ (black, *n = *32), *w;;GluClα*^*glc1*^*/+* (blue, *n = *34), *w;;GluClα*^*MI01156*^*/+*(green, *n = *30), *w;;GluClα*^*glc1/MI01156*^ (purple, *n = *18), and *w;;GluClα*^*glc1*^*/Df(3R)BSC636* (red, *n = *21) plotted as 30-min moving averages. **C-E** Total nighttime sleep **C**, number of night sleep episodes **D**, and mean duration of night sleep episodes **E** for each genotype. In **C**, one-way ANOVA with Dunnett’s *post hoc*, ns, *P > *0.05, ***P < 0.001; in **D** and **E,** Kruskal-Wallis followed by Dunn’s multiple comparisons test, ns, *P > *0.05, ***P < 0.001.

To confirm the role of GluClα in promoting nocturnal sleep, we obtained two hypomorphic alleles: *Mi{MIC}GluClα*^*MI01156*^, in which a transposon is inserted into the coding region of GluClα, and *GluClα*^*glc1*^, which contains the P298S missense mutation (Fig. 2A). Since all homozygous GluClα mutants were lethal, we generated *GluClα*^*MI01156/glc1*^ trans-heterozygotes by combining the two GluClα mutant alleles, *Mi{MIC}GluClα*^*MI01156*^ and *GluClα*^*glc1*^. These *GluClα*^*MI01156/glc1*^ flies exhibited a significant reduction in nocturnal sleep (Fig. 2B, C), a remarkable increase in nocturnal sleep episodes (Fig. 2D), and a reverse shortening in nocturnal sleep duration (Fig. 2E). Furthermore, similar sleep profiles were found in *GluClα*^*glc1*^-deficient flies, where the mutant allele was combined with *Df(3R)BSC636* (which lacks most of the GluClα gene) (Fig. 2B–E). These collective results suggest that GluClα is crucial for nocturnal sleep.

### GluClα Derived from VNC Neurons Facilitates Nocturnal Sleep

**Fig. 3:**
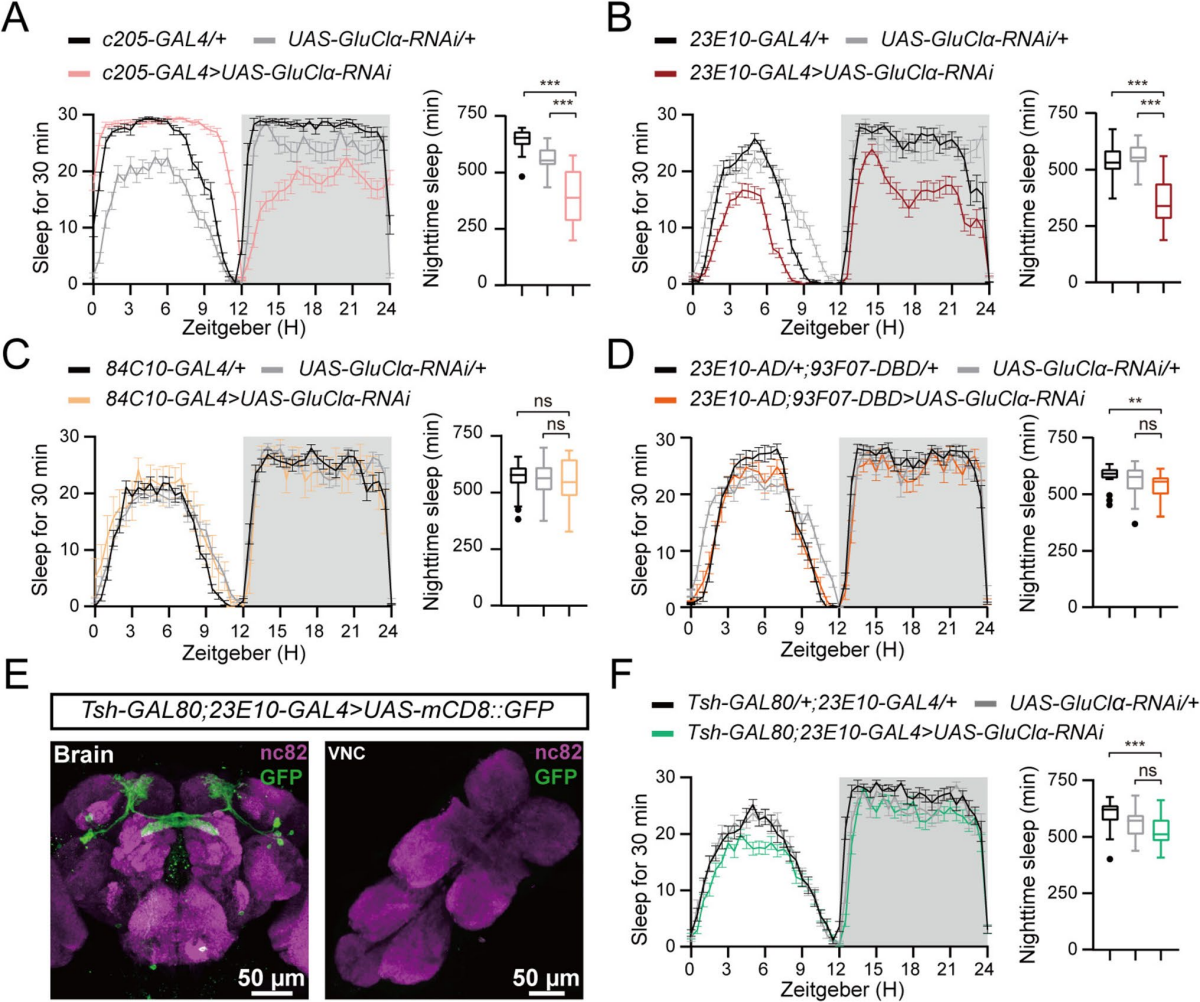
Knockdown of *GluClα* in the dFB does not affect sleep. **A** Sleep traces and quantification of nighttime sleep of *w;;c205-GAL4/+* (black, *n =* 30), *w;UAS-GluClα-RNAi/+* (light gray, *n = *29), and *w;UAS-GluClα-RNAi/+;c205-GAL4/+* (light red, *n = *49). **B** Sleep traces and quantification of nighttime sleep of *w;;23E10-GAL4/+* (black, *n = *31), *w;UAS-GluClα-RNAi/+* (light gray, *n = *29), and *w;UAS-GluClα-RNAi/+;23E10-GAL4/+* (dark red, *n =* 56). **C** Sleep traces and quantification of nighttime sleep of *w;;84C10-GAL4/+* (black, *n = *42), *w;UAS-GluClα-RNAi/+* (light gray, *n = *51), and *w;UAS-GluClα-RNAi/+;84C10-GAL4/+* (orange, *n = *11). **D** Sleep traces and quantification of nighttime sleep of *w;23E10-AD/+;97F07-DBD/+* (black, *n =* 31), *w;UAS-GluClα-RNAi/+* (light gray, *n = *29), and *w;23E10-AD/UAS-GluClα-RNAi;97F07-DBD/+* (light orange, *n =* 23). **E** The brain and VNC of an adult *Tsh-GAL80/+;23E10-GAL4/UAS-mCD8::GFP* fly double-stained with anti-GFP (green) and anti nc82 (purple); scale bars, 50 µm. **F** Sleep traces and quantification of nighttime sleep of *w;Tsh-GAL80/+;23E10-GAL4/+* (black, *n = *50), *w;UAS-GluClα-RNAi/+* (light gray, *n = *52), and *w;Tsh-GAL80/UAS-GluClα-RNAi;23E10-GAL4/+* (green, *n = *55). For **A**, **B**, **C**, **D**, **F**, one-way ANOVA with Dunnett’s post hoc, ns, *P > *0.05, ***P < 0.001.

To elucidate the anatomical requirement for GluClα in nocturnal sleep, we applied RNAi against GluClα using the Gal4/UAS (Upstream Activation Sequence) system. We tested > 20 Gal4 drivers with targeted expression in various regions of the nervous system (Table 2). RNAi against GluClα, using either the *23E10-GAL4* or the *c205-GAL4* driver, significantly reduced nocturnal sleep duration (Fig. 3A, B) without impacting sleep homeostasis (Fig. S1), whereas other drivers only elicited minor effects on nighttime sleep duration (Table 2). Given that both *23E10-GAL4* and *c205-GAL4* drivers label dorsal fan-shaped body (dFB) neurons [29–31] (Fig. S2A, B), we hypothesized that GluClα derived from dFB neurons was essential for nocturnal sleep. To test this, we knocked down GluClα in dFB neurons using other drivers. Surprisingly, RNAi against GluClα, using either the *84C10-GAL4* or *23E10-AD;93F07-DBD* driver [32] that label dFB neurons (Fig. S2C, D), had no discernible effect on nocturnal sleep (Fig. 3C, D). Recent studies have ruled out the involvement of dFB neurons labeled by *23E10-GAL4* in sleep regulation and have demonstrated that *tsh-GAL80* selectively targets sleep-promoting neurons in the VNC rather than the dFB [33]. Consistent with this, specifically knocking down GluClα in the dFB (*tsh-GAL80;23E10-GAL4>UAS-GluClα-RNAi*) failed to reduce nocturnal sleep (Fig. 3E, F). These findings suggest that GluClα expressed in dFB neurons is not involved in sleep regulation.

**Fig. 4:**
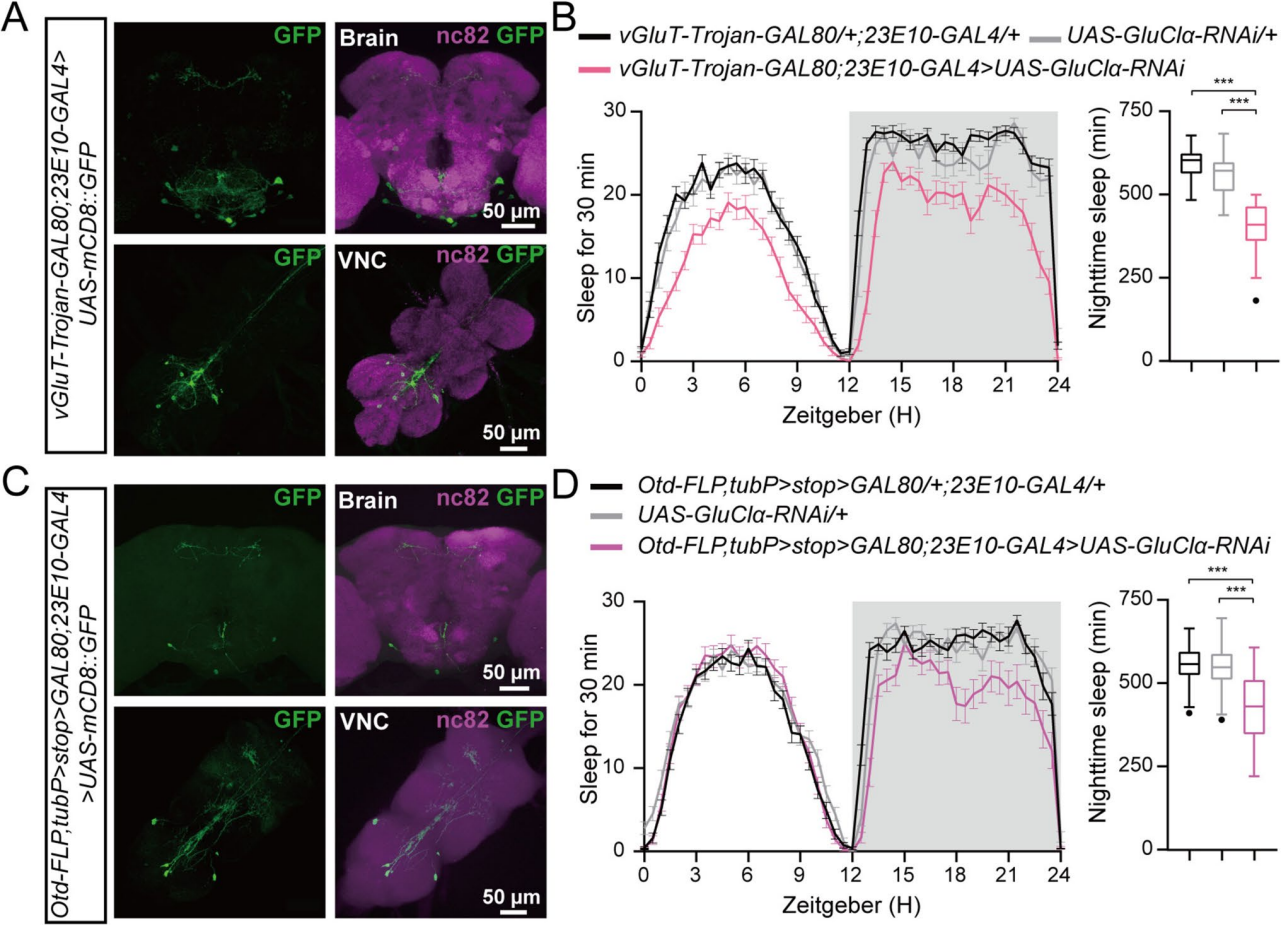
Reduced sleep after GluClα knockdown in 23E10^+^ neurons maps to the VNC. **A** The brain and VNC of an adult *w;vGluT-Trojan-GAL80/+;23E10-GAL4/UAS-mCD8::GFP* fly double-stained with anti-GFP (green) and anti nc82 (purple); scale bars, 50 µm. **B** Sleep traces and quantification of nighttime sleep of *w;vGluT-Trojan-GAL80;23E10-GAL4/+* (black, *n = *45), *w;UAS-GluClα-RNAi/+* (light gray, *n = *31), and *w;vGluT-Trojan-GAL80/UAS-GluClα-RNAi;23E10-GAL4/+* (pink, *n =* 46). **C** The brain and VNC of an adult *w;Otd-FLP,tubP>stoP > GAL80/+;23E10-GAL4/UAS-mCD8::GFP* fly double-stained with anti-GFP (green) and anti nc82 (purple); scale bars, 50 µm. **D** Sleep traces and quantification of nighttime sleep of *w;Otd-FLP,tubP>stoP > GAL80/+;23E10-GAL4/+* (black, *n = *52), *w;UAS-GluClα-RNAi/+* (light gray, *n = *52), and *w;Otd-FLP,tubP>stoP > GAL80/UAS-GluClα-RNAi;23E10-GAL4/+* (purple, *n = *36). For **B** and **D,** one-way ANOVA with Dunnett’s post hoc, ns, *P > *0.05, ***P < 0.001.

Next, we determined whether GluClα derived from VNC neurons promotes nocturnal sleep. Since dFB neurons have been reported to be glutamatergic [34, 35], *vGluT-trojan-GAL80* selectively targeted the dFB neurons rather than the VNC neurons (Fig. 4A). Moreover, we observed the co-localization of FB neurons marked by 23E10 with the vGluT-labeled glutamatergic neurons (Fig. S3), indicating that the *23E10-GAL4*-labeled-dFB neurons are indeed glutamatergic. Interestingly, specific RNAi against GluClα in the VNC (*vGluT-trojan-GAL80;23E10-GAL4>UAS-GluClα-RNAi*) significantly decreased nocturnal sleep (Fig. 4B). Furthermore, *Otd-FLP* has been reported to be selectively expressed in the central brain [36] but not in VNC neurons (Fig. 4C). Consistent with this, specific knockdown of GluClα in the VNC (*Otd-FLP;tubP>stoP > GAL80;23E10-GAL4>UAS-GluClα-RNAi*) decreased nocturnal sleep (Fig. 4D). Taken together, these results indicate that GluClα expressed by VNC neurons but not by dFB neurons, promotes nocturnal sleep.

### The Elimination of GluClα from Two Pairs of VNC Neurons Results in a Decrease in Nocturnal Sleep

**Fig. 5:**
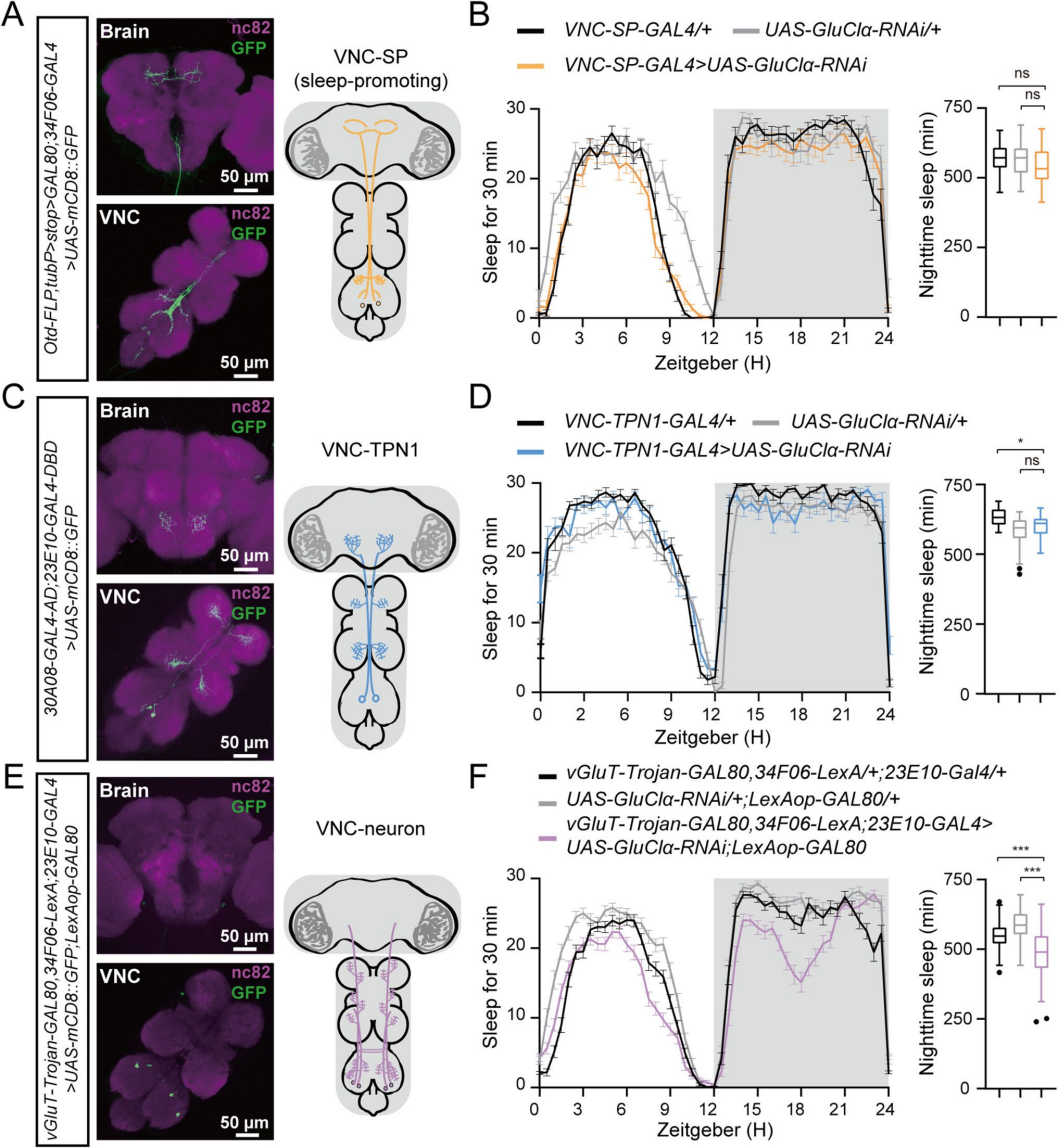
GluClα knockdown in two pairs of VNC neurons results in a decrease in nocturnal sleep. **A** Left: The brain and VNC of an adult *w;Otd-FLP,tubP>stoP > GAL80/+;34F06-GAL4/UAS-mCD8::GFP* fly double-stained with anti-GFP (green) and anti nc82 (purple); scale bars, 50 µm. Right: A projection model of VNC-SP neurons. **B** Sleep traces and quantification of nighttime sleep of *w;Otd-FLP,tubP>stoP > GAL80;34F06-GAL4/+* (black, *n = *29), *w;UAS-GluClα-RNAi/+* (light gray, *n =* 23), and *w;Otd-FLP,tubP>stoP > GAL80/UAS-GluClα-RNAi;34F06-GAL4/+* (orange, *n =* 31). **C** Left: The brain and VNC of an adult *w;30A08-AD/+;23E10-DBD/UAS-mCD8::GFP* fly double-stained with anti-GFP (green) and anti nc82 (purple); scale bars, 50 µm. Right: A projection model of VNC-TPN1 neurons. **D** Sleep traces and quantification of nighttime sleep of *w;30A08-AD/+;23E10-DBD/+* (black, *n = *22), *w;UAS-GluClα-RNAi/+* (light gray, *n = *42), and *w;30A08-AD/UAS-GluClα-RNAi;23E10-DBD/+* (blue, *n = *21). **E** Left: The brain and VNC of an adult *w; vGluT-Trojan-GAL80,34F06-LexA/UAS-mCD8::GFP;23E10-GAL4/LexAop-GAL80* fly double-stained with anti-GFP (green) and anti nc82 (purple); scale bars, 50 µm. Right: A projection model of VNC neurons. **F** Sleep traces and quantification of nighttime sleep of *w;vGluT-Trojan-GAL80,34F06-LexA;23E10-GAL4/+* (black, *n = *62), *w;UAS-GluClα-RNAi/+;LexAop-GAL80/+* (light gray, *n = *55), and *w; vGluT-Trojan-GAL80,34F06-LexA/UAS-GluClα-RNAi;23E10-GAL4/LexAop-GAL80* (purple, *n = *79). For **B**, **D**, and **F,** one-way ANOVA with Dunnett’s post hoc, ns, *P > *0.05, ***P < 0.001.

Our immunostaining results showed that *23E10-GAL4* labeled four pairs of neurons in the VNC region of adult flies (Fig. S2B). To elucidate the cellular mechanism by which GluClα facilitates nocturnal sleep, we sought to identify which neurons expressed GluClα that regulate this process. Recent studies have demonstrated that 23E10-labeled VNC neurons comprise a pair of sleep-promoting neurons (SP) and a pair of taste projection neurons 1 (TPN1) [37, 38]. Consequently, we initially tested whether knocking down GluClα specifically in either of these neuron types would impact nocturnal sleep. Our findings revealed that knocking down GluClα specifically in either VNC-SP neurons or TPN1 neurons did not influence nocturnal sleep (Fig. 5A–D). Our immunostaining results demonstrated that the *23E10-GAL4* driver within the VNC region not only outlines VNC-SP neurons and TPN1 neurons but also identifies two additional pairs of neurons, whose cell bodies are located in the metathoracic neuromere (Fig. S1B). Subsequently, we examined the repercussions of selectively reducing GluClα within these two pairs of neurons on fly sleep behavior. The outcomes demonstrated a significant reduction in nocturnal sleep upon the targeted inhibition of GluClα within these two pairs of VNC neurons (Fig. 5E, F).

Furthermore, knocking down GluClα in these two pairs of neurons resulted in an increase in nocturnal sleep bouts and shorter sleep bout durations in flies (Fig. S4), indicating that the glutamatergic signal indeed plays a pivotal role in consolidating nocturnal sleep through the expression of GluClα within these two pairs of neurons.

**Fig. 6:**
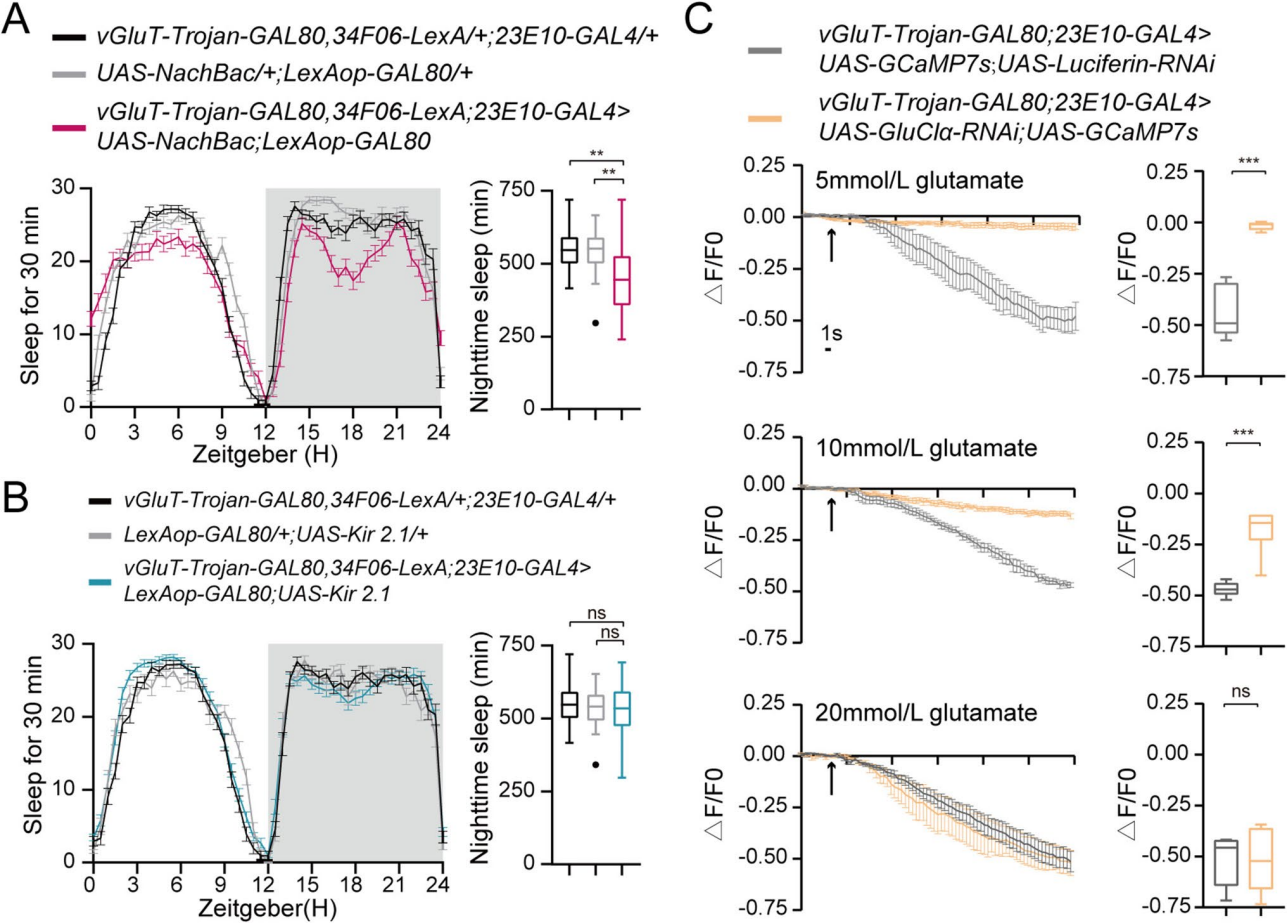
Glutamate inhibits the activity of 23E10^+^ VNC neurons. **A** Sleep traces and quantification of nighttime sleep of *w;vGluT-Trojan-GAL80,34F06-LexA;23E10-GAL4/+* (black, *n = *56), *w;UAS-NachBac/+;LexAop-GAL80* (light gray, *n =* 53), and *w; vGluT-Trojan-GAL80,34F06-LexA/UAS-NachBac;23E10-GAL4/LexAop-GAL80* (purple, *n = *70). **B** Sleep traces and quantification of nighttime sleep in *w;vGluT-Trojan-GAL80,34F06-LexA;23E10-GAL4/+* (black, *n = *56), *w;LexAop-GAL80/+;UAS-Kir2.1/+* (light gray, *n = *42) and *w;vGluT-Trojan-GAL80,34F06-LexA/LexAop-GAL80;23E10-GAL4/UAS-Kir2.1* (blue, *n = *93). In **A**, and **B,** one-way ANOVA with Dunnett’s post hoc, ns, *P >*0.05, ***P <0.001. **C** Mean GCaMP7s response traces of VNC neurons in *w;vGluT-Trojan-GAL80/UAS-GluClα-RNAi;23E10-GAL4/UAS-GCaMP7s* (orange; *n *= 6) and *w;vGluT-Trojan-GAL80/UAS-GCaMP7s 23E10-GAL4/UAS-Luciferin-RNAi* flies (gray; *n *= 6). Arrow, glutamate application. The results are shown as the mean ± SEM. Right panel, △F/F0 at 33 s of glutamate application for each genotype. Unpaired two-tailed Student’s *t*-test; ***P < 0.001.

To further confirm the involvement of the two identified pairs of VNC neurons in *Drosophila*'s nocturnal sleep, we induced activation by expressing NaChBac and subsequently monitored alterations in nocturnal sleep patterns. Activation of these neuronal pairs resulted in a significant reduction in nocturnal sleep compared to control groups (Fig. 6A). Conversely, silencing these same pairs *via* expression of the human inwardly-rectifying K^+^ channel Kir2.1 did not noticeably affect nocturnal sleep (Fig. 6B). These findings confirm that these two pairs of VNC neurons play a regulatory role in *Drosophila*'s nocturnal sleep, with GluClα depletion from these pairs leading to decreased nocturnal sleep.

### GluClα Mediates the Glutamate-gated Inhibitory input to the Wake-promoting Neurons in the VNC

GluClα, identified as a glutamate-gated chloride channel, provides inhibitory input to neurons. To investigate whether GluClα mediates this glutamate-gated inhibitory input *in vivo*, we directly measured the response mediated by GluClα in two pairs of 23E10^+^ VNC neurons. A picospritzer was applied to puff glutamate onto these neuron pairs, and this resulted in a rapid and robust reduction in [Ca^2+^] within the neurons (Fig. 6C and Videos S1, S2, S3). Conversely, only a slow reduction in [Ca^2+^] was induced in GluClα-depleted neurons upon glutamate puffing (Fig. 6C and Videos S1-S3). These findings suggest that the depletion of GluClα from these neuron pairs could potentially lead to their hyperactivity, thereby reducing nocturnal sleep.

**Fig. 7:**
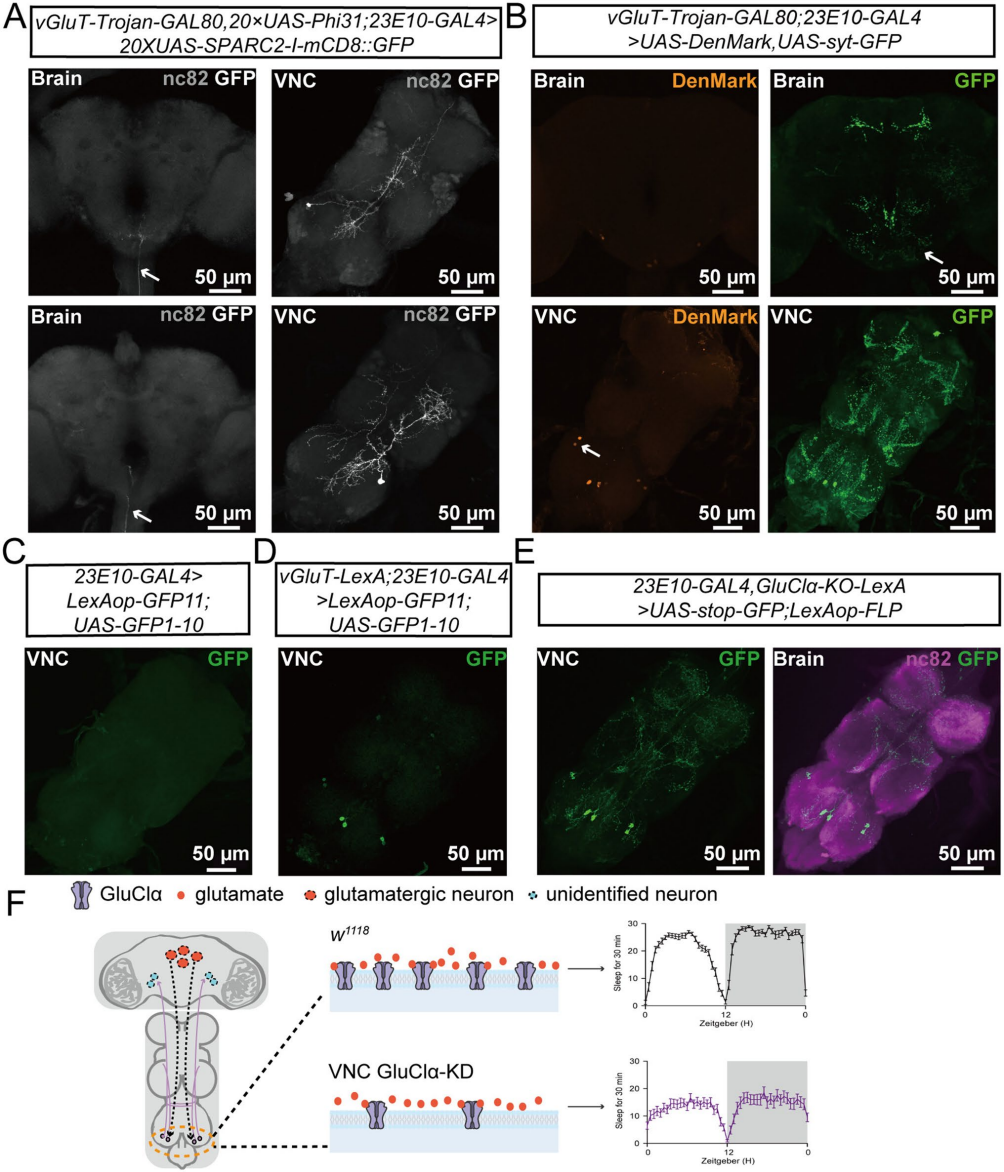
Morphology of the 23E10^+^ VNC neurons and their synaptic contact with glutamatergic neurons at the soma. **A** *SPARC2-mCD8::GFP* expression (white) in target neurons counterstained with anti-nc82 (gray), which selectively labels individual VNC neurons; scale bars, 50 µm. **B** *vGluT-Trojan-GAL80/+;23E10-GAL4/+* drives the expression of DenMark and Syt::GFP in the adult brain and VNC. DenMark expression is restricted to around the soma, whereas Syt::GFP is found in the brain and VNC; scale bars, 50 µm. **C**, **D** GRASP signals in control (**C**) and between glutamatergic neurons and VNC neurons (**D**); scale bars: 50 µm. **E** VNC of adult *UAS-FRT-stop-FRT-mCD8-GFP/+;23E10-GAL4,GluClα-LexA/lexAop-Flp* fly double-stained with anti-GFP (green) and anti-nc82 (purple). mCD8-GFP is expressed under the *23E10-GAL4* driver only after the transcriptional stop cassette (*>stoP >*) is removed from *UAS>stoP > mCD8-GFP* by flippase, which is expressed by *GluClα*-LexA; scale bars,50 µm. **F** Model of the VNC-neuron regulation of sleep by GluClα.

In order to elucidate the mechanism by which GluClα, expressed in these two pairs of VNC neurons, regulates nocturnal sleep in *Drosophila*, we first discerned their morphology by sparse-labeling analysis [39]. The results showed that the somas of these neurons are situated in the metathoracic neuromere, and their axonal projection areas encompassed the mesothoracic neuromere, extending to the subesophageal ganglion (SOG) of the brain (Fig. 7A).

Next, to identify the presynaptic and postsynaptic sites. We expressed synaptotagmin-GFP as a presynaptic marker and DenMark as a postsynaptic marker in the 23E10-labeled VNC neurons. The expression of synaptotagmin-GFP revealed that these two pairs of VNC neurons send axons to both the mesothoracic neuromere in the VNC and the SOG in the brain (Fig. 7B). Conversely, DenMark was only detected in the soma of VNC neurons (Fig. 7B). To determine whether these two pairs of 23E10^+^ VNC neurons form direct synaptic connections with glutamatergic neurons, we applied the technique of activity-dependent GFP reconstitution across synaptic partners (GRASP) [40]. Our findings revealed that indeed, in the brain, 23E10^+^ neurons establish direct synaptic connections with glutamatergic neurons in the dFB region (Fig. S5A, B). Moreover, within the VNC, as compared to the control group (Fig. 7C), it was equally evident that reconstructed GFP signals can be observed in the soma of these two neuronal pairs (Fig. 7D). Through the implementation of a binary expression cross strategy, we further corroborated the expression of GluClα in these 23E10^+^ neurons (Figs 7E and S5C). These findings suggest that these two pairs of 23E10^+^ VNC neurons establish direct synaptic connections with glutamatergic neurons, thereby enabling inhibitory inputs to wake-promoting neurons located in the VNC mediated by GluClα.

## Discussion

Sleep is an evolutionarily conserved behavior from worms to humans [1, 2]. *Drosophila* is an ideal model organism that allows for the dissection of the underlying mechanisms that regulate sleep, thereby providing insights into the molecular, cellular, and neural circuit levels of sleep regulation. While glutamate is generally considered a wake-promoting neurotransmitter in mammals, its role in *Drosophila* sleep remains unclear. In this study, we identified two pairs of VNC neurons that express GluClα and receive glutamate-gated inhibitory input to promote sleep. Our findings suggest that GluClα enhances nocturnal sleep by mediating the glutamate-gated inhibitory input to these VNC neurons, thereby providing new insights into the mechanism of sleep promotion in *Drosophila*.

### Glutamate Signaling Regulates Sleep

Glutamate serves as the primary excitatory neurotransmitter in both mammals and *Drosophila*, and its role as a wakefulness-promoting neurotransmitter is well-established in mammals. Studies have demonstrated that glutamate concentrations in the prefrontal cortex and motor cortex increase progressively during wakefulness and REM periods [41]. Conversely, the rate of decrease in glutamate is positively correlated with sleep intensity during NREM periods [41]. The paraventricular thalamus (PVT) in mice plays a crucial role in regulating wakefulness; glutamatergic neurons in this region exhibit high activity during wakefulness [42]. Inhibition of PVT neuronal activity results in a reduction in mouse wakefulness time, while activation of these neurons induces a transition from sleep to wakefulness [42]. However, the potential roles of glutamate signaling in *Drosophila* sleep are less well understood, with some studies yielding contradictory results. Single-cell transcriptome data suggest that ~24% of the adult fly brain neurons are glutamatergic [43]. Activation of these neurons significantly reduces fruit sleep time, suggesting that glutamate may function as a wakefulness-promoting neurotransmitter in *Drosophila* [18]. However, other studies indicate that glutamate acts as a sleep-promoting neurotransmitter, enhanced presynaptic glutamate release promoting sleep through over-activation of postsynaptic N-methyl D- aspartate receptors (NMDARs) [44]. Consistent with this, depletion of NMDAR1 or application of NMADR antagonists results in a reduction in sleep time [20]. In this study, we demonstrated that neuronal cell-specific knockdown of vGluT reduces nocturnal sleep. Despite being the primary excitatory neurotransmitter, the exact roles of glutamate depend on its specific location and receptor type. Within the glutamatergic system, glutamate can trigger excitatory input by activating glutamate receptors on the postsynaptic membrane of glutamatergic neurons [21]. In the GABAergic system, glutamate can increase GABAergic inhibitory tone across the circuit by activating presynaptic glutamate receptors [22]. Furthermore, although glutamate is typically an excitatory neurotransmitter in flies, it can also be inhibitory in the CNS, acting mainly through the GluClα [24, 45–47].

GluClα, a member of the ligand-gated ion channel superfamily, has been demonstrated to induce a rapidly-activated inward membrane current in *Xenopus laevis* oocytes transfected with GluClα mRNA through *in vitro* electrophysiological recording [48, 49]. In this study, we present evidence that either *GluClα* mutant flies or depletion of GluClα VNC neurons leads to a reduction in sleep time. Prior electrophysiological recordings have revealed that *GluClα*^*glc1*^ mutants exhibit diminished sensitivity to glutamate [49]. Interestingly, our research indicates that *GluClα*^*glc1*^ heterozygous mutants display a decrease in nighttime sleep. These findings further substantiate the role of GluClα in regulating sleep. Our GRASP data underscore that VNC neurons are recipients of the upstream glutamate signal, and our Ca^2+^ imaging revealed that the application of glutamate rapidly inhibits VNC neurons. These findings suggest that VNC neurons receive inhibitory glutamate inputs *via* GluClα.

The GluClα channel in fruit flies is homologous to human GLRA1 (glycine receptor alpha 1) and both belong to the Cys-loop ligand-gated ion channel family [50]. The intimate evolutionary relationship between GluClαs and glycine receptors (GlyRs) may manifest in glutamate's capacity to allosterically potentiate GlyR-gated chloride Cl^–^ [51]. GluClα serves as an inhibitory channel, triggering Cl^–^ influx and hyperpolarization upon activation by glutamate [48]. In mammals, glycine receptors play a similar inhibitory role in synaptic transmission [52], particularly in the spinal cord and brainstem, aiding in mediating crucial inhibitory signals vital for various physiological processes [53, 54]. Notably, the pairs of neurons expressing GluClα that we have identified as regulating sleep are situated within the fruit fly's VNC, analogous to the spinal cord in vertebrates. In the brain, glycine may facilitate excitatory transmission through an allosteric activation of the NMDA receptor [55]. In addition, there are studies indicating that exogenous glycine can stimulate the release of D-aspartate, partly through the activation of the glycine transporters GlyT1 and GlyT2, and that D-aspartate can stimulate glycine release in a process sensitive to glutamate transporter blockers [56], which indicates that there may be a form of crosstalk between glycine and glutamate through the activation of their transporters in hippocampal nerve terminals. These studies reflect the diverse functions of these neurotransmitters in distinct regions of the nervous system. Nonetheless, our exploration of GluClα in fruit flies has illuminated the workings of mammalian glycine receptors, fostering the advancement of therapeutic strategies for disorders associated with these receptors.

### VNC Neurons are Involved in Sleep Regulation

Research on sleep regulation in *Drosophila* predominantly focuses on the rhythmic neurons and the central nervous system within the brain [57]. Prior studies have proposed that the driving factors of sleep homeostasis may exert their behavioral effects by acting on neurons outside the brain, with PPK (pickpocket) neurons in the legs serving as wake effectors, converting accumulated information into sleep needs [58]. Recent studies have shown that some VNC neurons are also implicated in sleep regulation [33, 37]. These studies found that two *23E10-GAL4*-labeled neurons located in the VNC play a role in sleep regulation. The activation of these neurons increases the wake threshold and enhances sleep in fruit flies, confirming that VNC neurons promote sleep by releasing acetylcholine when activated [37]. In this study, we demonstrate that the activation of VNC neurons results in reduced nighttime sleep, and the knockdown of GluClα in these neurons recapitulates the reduced nighttime sleep phenotype. We further demonstrated that these neurons receive inhibitory glutamate signaling. The expression of the epitope-tagged presynaptic marker synaptotagmin-GFP revealed that these VNC neurons send axons to the subesophageal ganglion (SOG), while the expressed postsynaptic marker DenMark was only detected in their somata. These findings suggest that these neurons receive glutamate signaling within the VNC and transmit the output signal to the SOG. Although the exact source of the glutamate signal remains unknown, we identified some glutamatergic neurons that send their axons into the VNC. This suggests that these VNC neurons may receive and integrate glutamate inputs from peripheral or brain sources and then transmit this information back to the brain. The origin of the glutamate signals and the output signals of these VNC neurons require further investigation. In summary, our study identifies two pairs of GluClα-expressing neurons in the *Drosophila* VNC that promote sleep. They receive and integrate inhibitory glutamatergic signals from the periphery and transmit sleep information to the brain. This discovery not only expands our understanding of the function of the glutamate neurotransmitter but also provides new perspectives and a theoretical basis for further exploration of complex sleep regulatory networks.

## Supplementary Information

Below is the link to the electronic supplementary material.

Supplementary file1 (PDF 1877 KB)

Supplementary file2 (MP4 3899 KB)

Supplementary file3 (MP4 3224 KB)

Supplementary file4 (MP4 4635 KB)
