# Supplementary material for: The Glutamate-gated Chloride Channel Facilitates Sleep by Enhancing the Excitability of Two Pairs of Neurons in the Ventral Nerve Cord of Drosophila
Source: Neurosci Bull. 2025 Apr 30;41(10):1729–42. doi: 10.1007/s12264-025-01397-1 (PMC12494514; doi:10.1007/s12264-025-01397-1)
Supplement: Supplementary file 1 — Supplementary file1 (PDF 1877 KB) [file 12264_2025_1397_MOESM1_ESM.pdf]

Supplementary Materials

Supplementary Figures and Legends

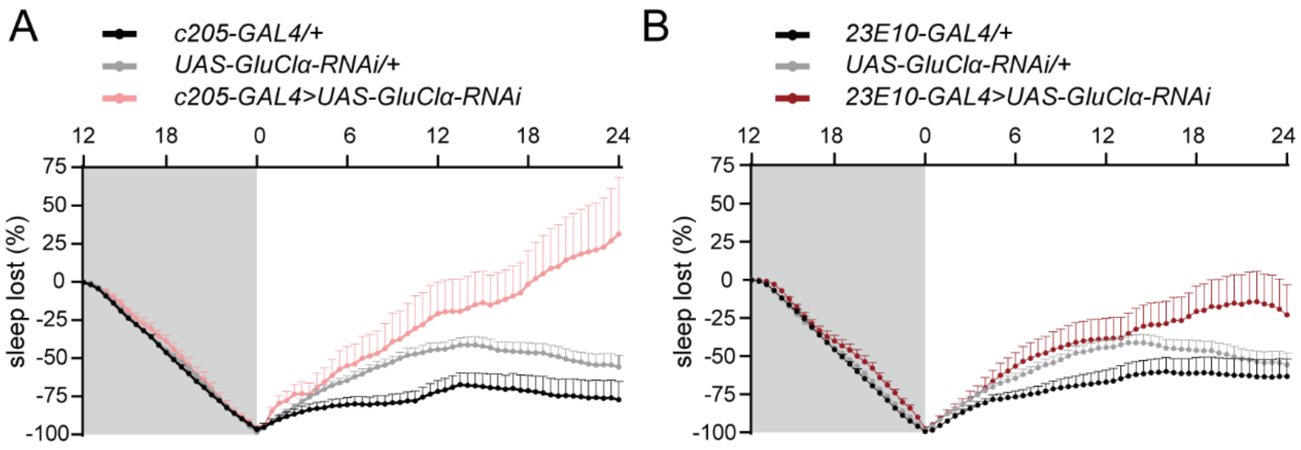

Fig. S1 The absence of *GluClα* does not affect sleep homeostasis in *Drosophil*a (Related to Fig. 3).

A, **B** Cumulative sleep is lost during 12 h of sleep deprivation and regained during subsequent recovery for 24 h in different genotypes. *w;;c205-GAL4/+* (black, *n* = 11); *w;UAS-GluClα-RNAi/+*(light grey, *n* = 20); *w;UAS-GluClα-RNAi/+;c205-GAL4/+* (light red, *n* = 13); *w;;23E10-GAL4/+* (black, *n* = 15); *w;UAS-GluClα-RNAi/+* (light grey, *n* = 20); *w;UAS-GluClα-RNAi/+;23E10-GAL4/+* (dark red, *n* = 17).

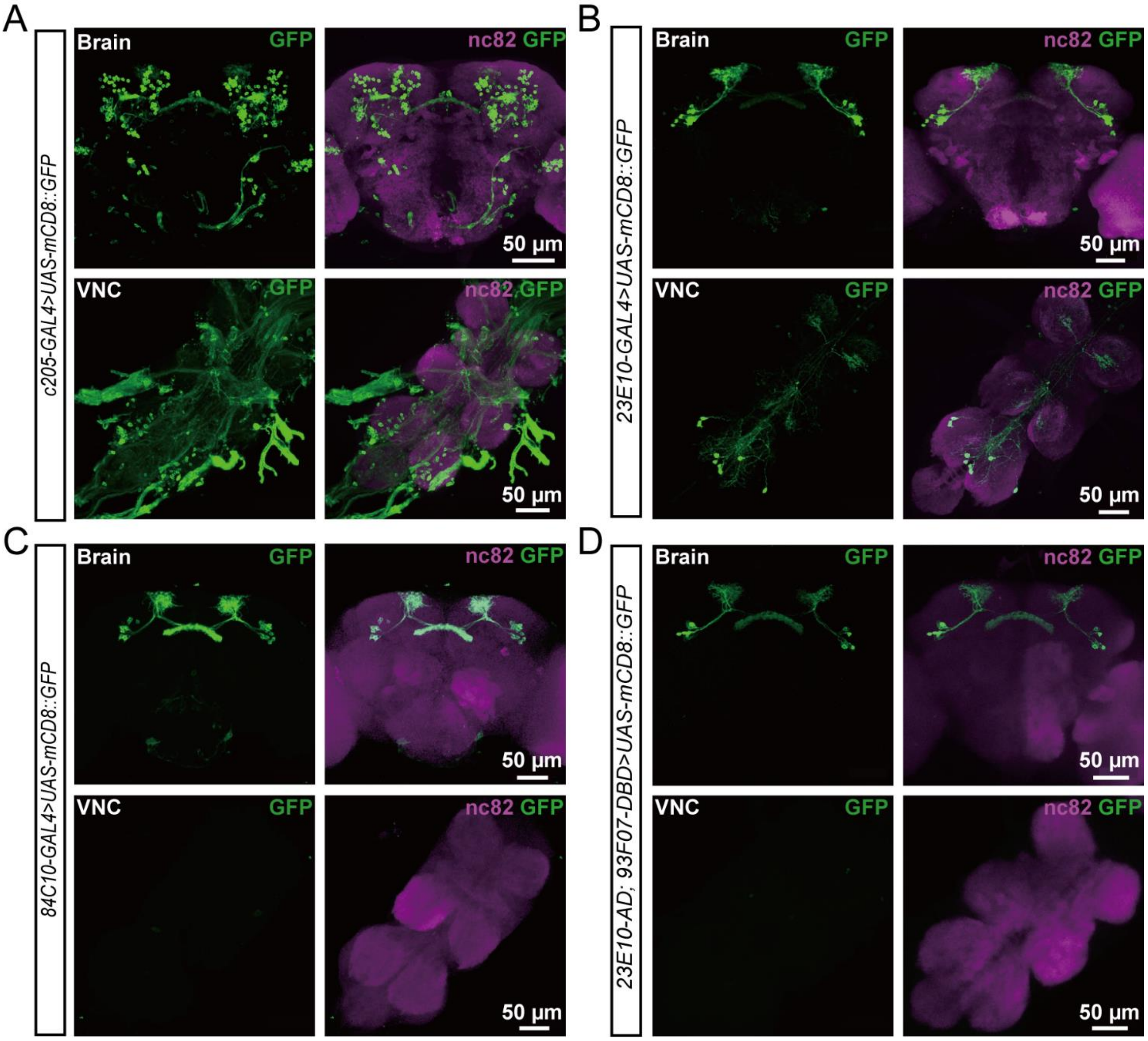

Fig. S2 The expression pattern in a dorsal fan-shaped body (dFB)-related fly (Related to Fig. 3). **A-D** The brain and VNC of an adult fly double stained with anti-GFP (green) and anti nc82 (purple). The genotypes are *w;;c205-GAL4/UAS-mCD8::GFP* (**A**), *w;;23E10-GAL4,UAS-mCD8::GFP/23E10-GAL4,UAS-mCD8::GFP* (**B**), *w;;84C10-GAL4/UAS-mCD8::GFP* (**C**), and *w; 23E10-AD/+;97F07-DBD/UAS-mCD8::GFP* (**D**); scale bars, 50 μm.

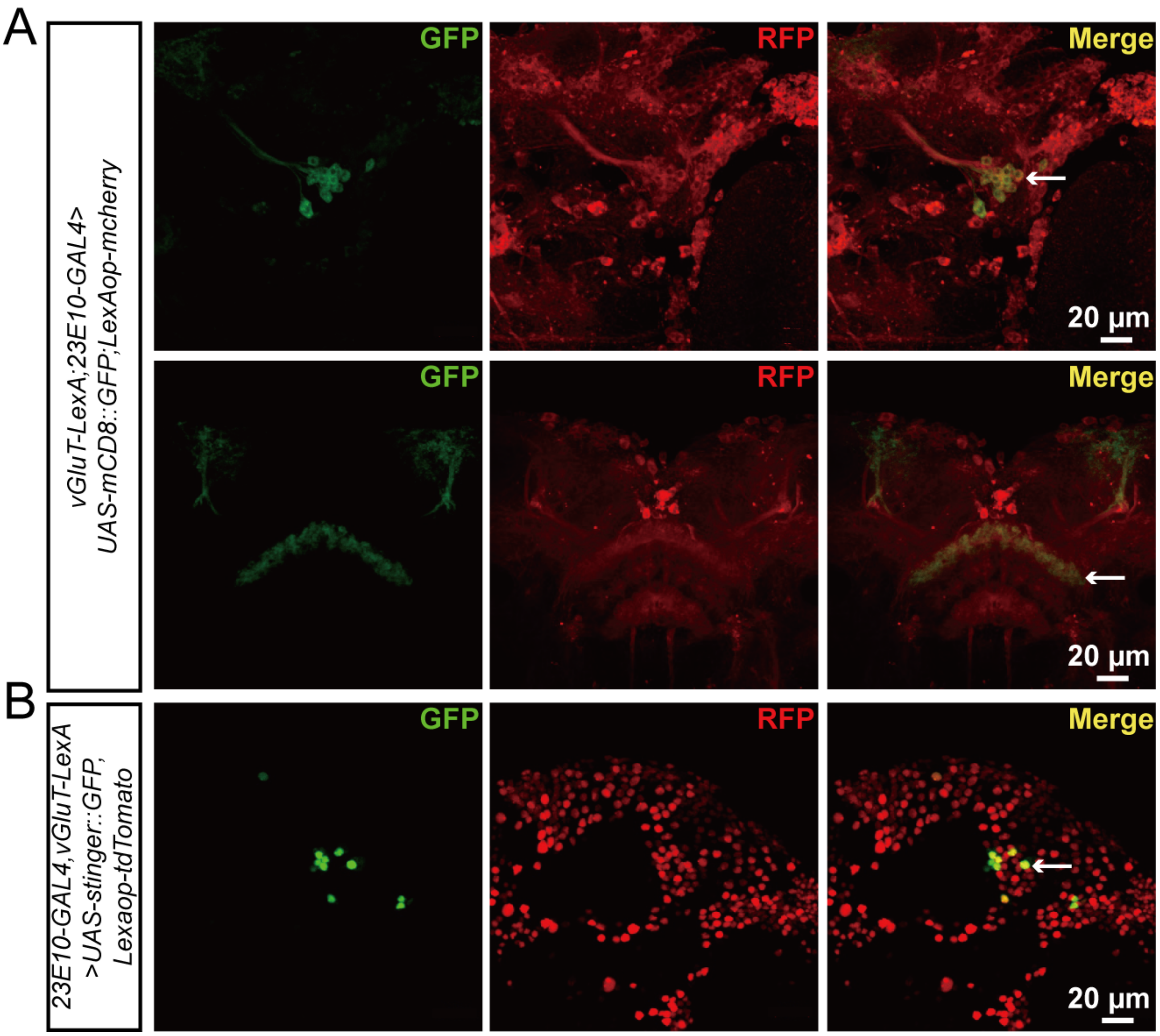

Fig. S3 23E10-positive neurons expressing glutamate (Related to Fig. 4). **A** 23E10-positive neurons co-localize with glutamate: UAS-GFP driven by 23E10-GAL4 overlaps with vGluT-driven LexAop-mcherry; all 23E10-positive neurons express glutamate. The genotypes are *vGluT-LexA/UAS-mCD8::GFP;23E10-GAL4/LexAop-mcherry*; scale bars, 20 µm. **B** 23E10-positive neurons labeled by nuclear localization of GFP, and vGluT-expressing neurons are labeled by tdTomato. Note overlap of 23E10-positive neurons with glutamatergic neuronal immunostaining; scale bar, 20 µm.

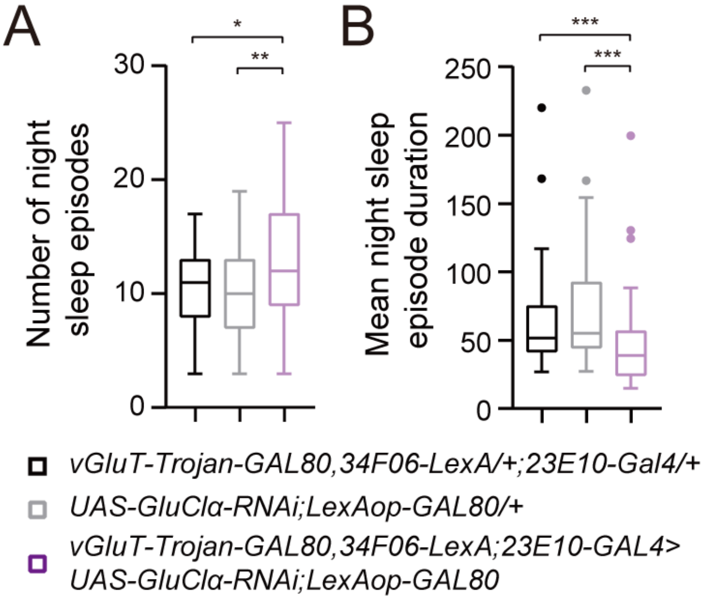

Fig. S4 Fragmented nighttime sleep in *Drosophila* lacking GluClα (Related to Fig. 5). **A**, **B** Number of night sleep episodes (**A**), and mean night sleep episode duration (**B**) for *w;vGluT-Trojan-GAL80,34F06-LexA;23E10-GAL4/+* (black, n = 62), *w;UAS-GluClα-RNAi/+;LexAop-GAL80/+* (light grey, n = 55), and *w;vGluT-Trojan-GAL80,34F06-LexA/UAS-GluClα-RNAi;23E10-GAL4/LexAop-GAL80* (purple, n = 79). In **A** and **B,** Kruskal-Wallis followed by Dunn's multiple comparisons test, ns, P >0.05, *P < 0.05, **P <0.01, ***P < 0.001.

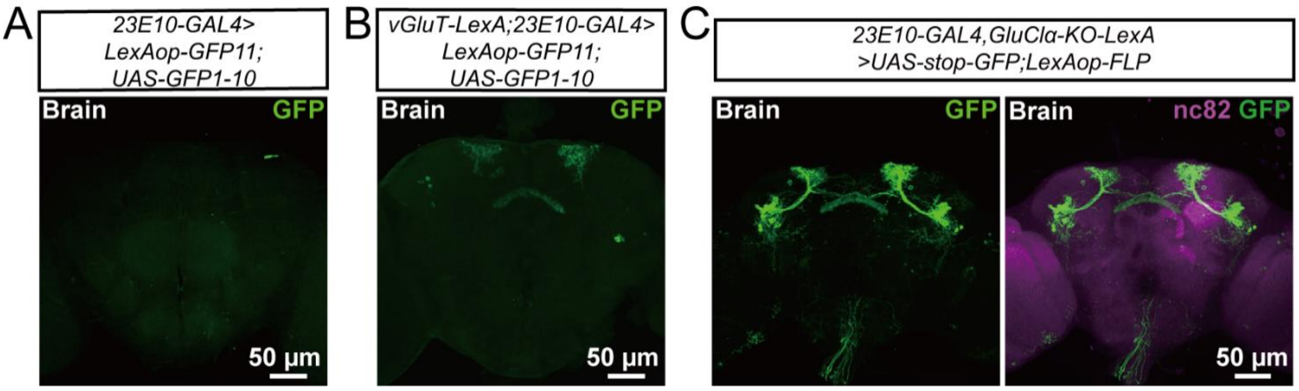

Fig. S5 Neurons in the brain express *GluClα* and receive glutamate signals (Related to Fig. 7). **A**, **B** GRASP signals in control brain (**A**) and between glutamatergic neurons and 23E10-labeled neurons (**B**). **C** Brain of adult *UAS-FRT-stop-FRT-mCD8-GFP/+;23E10-GAL4,GluClα-LexA/lexAop-Flp* fly double-stained with anti-GFP (green) and anti-nc82 (purple). mCD8-GFP is expressed under the *23E10-GAL4* driver only after the transcriptional stop cassette (>sto*P*>) is removed from *UAS>stoP > mCD8-GFP* by flippase, which is expressed by GluClα-LexA. Scale bars, 50 μm.

S1 Video. Recording of GluClα-positive neuronal responses to 5 mM glutamate (Related to Fig. 6C). The genotypes of flies are annotated at the top left of the video.

S2 Video. Recording of GluClα-positive neuronal responses to 10 mM glutamate (Related to Fig. 6C). The genotypes of flies are annotated at the top left of the video.

S3 Video. Recording of GluClα-positive neuronal responses to 20 mM glutamate (Related to Fig. 6C). The genotypes of flies are annotated at the top left of the video.

Supplementary Tables

Table 1 RNAi lines used for GluR screening [1].

CG Number	Name	Gene ID	Locus	RNAi Line
mGluR				
CG11144	*mGluRA*	43838	102F1-102F3	THU5288
Kainate				
CG6992	*GluRIIA*	33788	25E6-25E6	THU2659
CG7234	*GluRIIB*	33789	25E6-25E6	THU3089
CG4226	*GluRIIC*	33275	21E2-21E2	THU2049
CG18039	*GluRIID*	44483	92F4-92F4	THU2151
CG31201	*GluRIIE*	318623	92F4-92F4	THU3986
CG8681	*clumsy*	35394	39B2-39B3	BL28351
CG3822	*KaiR1D*	42473	93A2-93A2	THU3982
CG5621	*Grik*	42476	93A2-93A3	THU3979
CG9935	*Ekar*	43806	102D1-102D1	THU3080
CG11155		43822	102F8-102F8	THU3285
AMPA				
CG8442	*GluRIA*	38742	65C1	THU5238
CG4481	*GluRIB*	44484	67A4-67A6	BL67843
NMDA				
CG2902	*Nmdar1*	3345094	83A6-83A7	THU2118
CG33513	*Nmdar2*	31107	2B1	THU5862

GluClα				
CG7535	*glc/GluCl*α	42350	92B1-92B2	BL53356

Reference

1. Hu W, Wang T, Wang X, Han J. Ih channels control feedback regulation from amacrine cells to photoreceptors. PLoS Biol 2015, 13: e1002115.

Table 2 Properties of the GAL4 lines used in the behavioral experiments.

Name	Nearest or related gene	Expression pattern	Genotype	Night sleep (mean±SEM)	Difference	Reference
repo-GAL4	repo	glial cell	repo-GAL4/+	612.9±6.9	ns	[1]
			repo-GAL4/UAS-GluClα-RNAi	574.8±7.474		
R6-GAL4	n.d.	s-LNv	R6-GAL4/+	575±10.62	ns	[2, 3]
			R6-GAL4/UAS-GluClα-RNAi	469.8±12.22		
78G01-GAL4	SPR (sex peptide receptor)	l-LNv	78G01-GAL4/+	546±9.967	ns	[4, 5]
			78G01-GAL4/UAS-GluClα-RNAi	553.1±10.55		
c929-GAL4	n.d.	Peptidergic neurons l-LNv	c929-GAL4/+	619.5±8.887	*	[6, 7]
			c929-GAL4/UAS-GluClα-RNAi	516.6±12.5		
per-GAL4	per	Photoreceptor neurons LN period neurons	per-GAL4/+	635.3±8.297	ns	
			per-GAL4/UAS-GluClα-RNAi	603.3±7.639		
Clk	Clk	LN period	Clk 856-GAL4/+	624.8±9.631	*	[8]

856-GAL4		neurons DN period neurons LNv neurons	Clk 856-GAL4/UAS-GluClα-RNAi	507.4±11.94		
Clk4.1M-GAL4	Clk	DN1 neurons	Clk4.1M-GAL4/+	596.8±9.852	ns	[9, 10]
			Clk4.1M-GAL4 /UAS-GluClα-RNAi	550±13.96		
54D11-GAL4	Rab interacting lysosomal protein like	LNd neurons	54D11-GAL4/+	593.7±16.51	ns	[4, 5]
			54D11-GAL4/UAS-GluClα-RNAi	647.2±10.24		
CNMa-GAL4	CNMa	DN neurons SOG	CNMa-GAL4/+	601.8±20.25	ns	[11]
			CNMa-GAL4/UAS-GluClα-RNAi	645.1±11.53		
58H05-	loaf (lost	EB	58H05-GAL4/+	512.7±11.91	*	[4, 5]

GAL4	and found)		58H05-GAL4/UAS-GluClα-RNAi	506.8±18.41		
OK107-GAL4	n.d.	MB	OK107-GAL4/+	602±12.78	ns	[12]
			OK107-GAL4/UAS-GluClα-RNAi	598±11.67		
84A01-GAL4	bon (bonus)	FB	84A01-GAL4/+	496±19.21	*	[4, 5]
			84A01-GAL4/UAS-GluClα-RNAi	477.2±24.85		
104y-GAL4	n.d.	FB	104y-GAL4/+	590±12.89	ns	[13]
			104y-GAL4/UAS-GluClα-RNAi	508.9±20.84		
Dh44-GAL4	Dh44 (Diuretic hormone 44)	PI	Dh44-GAL4/+	609.3±11.41	ns	[14, 15]
			Dh44-GAL4/UAS-GluClα-RNAi	538.3±16.51		
Lk-GAL4	Lk (Leucokinin)	n.d.	Lk-GAL4/+	549.8±19.31	ns	
			Lk-GAL4/UAS-GluClα-RNAi	485.7±13.6		
TH-D-		PPM PPL	TH-D-GAL4/+	626.1±7.621	ns	[16, 17]

GAL4			TH-D-GAL4/UAS-GluClα-RNAi	572.4±9.957		
Hug-GAL4	Hug (Hugin)	Subesophageal zone	Hug-GAL4/+	580.4±13.17	ns	[18]
			Hug-GAL4/UAS-GluClα-RNAi	502.2±19.67		
dlip2-GAL4	dlip2 (Dorsal interacting protein 2)	PI	dlip2-GAL4/+	587.3±24.46	ns	[15]

n.d., no definition

**P* <0.05, ***P* <0.01, ****P* <0.001; ns, no significant difference, *P* >0.05.

References

1. Awasaki T, Lai SL, Ito K, Lee T. Organization and postembryonic development of glial cells in the adult central brain of Drosophila. J Neurosci 2008, 28: 13742-13753.

2. Zhao X, Yang X, Lv P, Xu Y, Wang X, Zhao Z, *et al.* Polycomb regulates circadian rhythms in Drosophila in clock neurons. Life Sci Alliance 2024, 7.

3. Helfrich-Forster C, Shafer OT, Wulbeck C, Grieshaber E, Rieger D, Taghert P. Development and morphology of the clock-gene-expressing lateral neurons of Drosophila melanogaster. J Comp Neurol 2007, 500: 47-70.

4. Jenett A, Rubin GM, Ngo TT, Shepherd D, Murphy C, Dionne H, *et al.* A GAL4-driver line resource for Drosophila neurobiology. Cell Rep 2012, 2: 991-1001.

5. Pfeiffer BD, Jenett A, Hammonds AS, Ngo TT, Misra S, Murphy C*, et al.* Tools for neuroanatomy and neurogenetics in Drosophila. Proc Natl Acad Sci U S A 2008, 105: 9715-9720.

6. Shang Y, Griffith LC, Rosbash M. Light-arousal and circadian photoreception circuits intersect at the large PDF cells of the Drosophila brain. Proc Natl Acad Sci U S A 2008, 105: 19587-19594.

7. Taghert PH, Hewes RS, Park JH, O'Brien MA, Han M, Peck ME. Multiple amidated neuropeptides are required for normal circadian locomotor rhythms in Drosophila. J Neurosci 2001, 21: 6673-6686.

8. Gummadova JO, Coutts GA, Glossop NR. Analysis of the Drosophila Clock promoter reveals heterogeneity in expression between subgroups of central oscillator cells and identifies a novel enhancer region. J Biol Rhythms 2009, 24: 353-367.

9. Yadlapalli S, Jiang C, Bahle A, Reddy P, Meyhofer E, Shafer OT. Circadian clock neurons constantly monitor environmental temperature to set sleep timing. Nature 2018, 555: 98-102.

10. Zhang Y, Liu Y, Bilodeau-Wentworth D, Hardin PE, Emery P. Light and temperature control the contribution of specific DN1 neurons to Drosophila circadian behavior. Curr Biol 2010, 20: 600-605.

11. Deng B, Li Q, Liu X, Cao Y, Li B, Qian Y*, et al.* Chemoconnectomics: Mapping Chemical Transmission in Drosophila. Neuron 2019, 101: 876-893 e874.

12. Connolly JB, Roberts IJ, Armstrong JD, Kaiser K, Forte M, Tully T*, et al.* Associative learning disrupted by impaired Gs signaling in Drosophila mushroom bodies. Science 1996, 274: 2104-2107.

13. Donlea JM, Thimgan MS, Suzuki Y, Gottschalk L, Shaw PJ. Inducing sleep by remote control facilitates memory consolidation in Drosophila. Science 2011, 332: 1571-1576.

14. Ohhara Y, Kobayashi S, Yamakawa-Kobayashi K, Yamanaka N. Adult-specific insulin-producing neurons in Drosophila melanogaster. J Comp Neurol 2018, 526: 1351-1367.

15. Cavanaugh DJ, Geratowski JD, Wooltorton JR, Spaethling JM, Hector CE, Zheng X*, et al.*

Identification of a circadian output circuit for rest:activity rhythms in Drosophila. Cell 2014, 157: 689-701.
16. Kondo S, Takahashi T, Yamagata N, Imanishi Y, Katow H, Hiramatsu S*, et al.* Neurochemical Organization of the Drosophila Brain Visualized by Endogenously Tagged Neurotransmitter Receptors. Cell Rep 2020, 30: 284-297 e285.
17. Liu Q, Liu S, Kodama L, Driscoll MR, Wu MN. Two dopaminergic neurons signal to the dorsal fan-shaped body to promote wakefulness in Drosophila. Curr Biol 2012, 22: 2114-2123.
18. King AN, Barber AF, Smith AE, Dreyer AP, Sitaraman D, Nitabach MN*, et al.* A Peptidergic Circuit Links the Circadian Clock to Locomotor Activity. Curr Biol 2017, 27: 1915-1927 e1915.

Table 3. *Drosophila* Resource Table.

Reagent type (species) or resource	Designation	Source or reference	Identifiers
Genetic reagent (*D. melanogaster*)	*nSyb-GAL4*	Bloomington *Drosophila* Stock Center	RRID:BDSC-51635
Genetic reagent (*D. melanogaster*)	*UAS-GluClα-RNAi*	Bloomington *Drosophila* Stock Center	RRID:BDSC-53356
Genetic reagent (*D. melanogaster*)	*R6-GAL4*	Gift from Paul Taghert lab (Washington University, St. Louis, USA)	N/A
Genetic reagent (*D. melanogaster*)	*C929-GAL4*	Bloomington *Drosophila* Stock Center	RRID:BDSC-25373
Genetic reagent (*D. melanogaster*)	*vGluT-LexA*	Gift from Yufeng Pan (Southeast University, Nanjing, China)	N/A
Genetic reagent (*D. melanogaster*)	*UAS-FRT-stop-FRT-GFP*	Bloomington *Drosophila* Stock Center	RRID: BDSC_30125
Genetic reagent (*D. melanogaster*)	*LexAop-FLP*	Bloomington *Drosophila* Stock Center	RRID:BDSC-55819
Genetic reagent (*D. melanogaster*)	*UAS-Luciferin*	Gift from Pengyu Gu (Sir Run Run Shaw Hospital, Zhejiang, China)	N/A

Genetic reagent (*D. melanogaster*)	*repo-GAL4*	Bloomington *Drosophila* Stock Center	RRID:BDSC-7415
Genetic reagent (*D. melanogaster*)	*OK 107-GAL4*	Kyoto Stock Center	DGRC-106098
Genetic reagent (*D. melanogaster*)	*c205-GAL4*	Bloomington *Drosophila* Stock Center	RRID:BDSC-30825
Genetic reagent (*D. melanogaster*)	*23E10-GAL4*	Bloomington *Drosophila* Stock Center	RRID:BDSC-49032
Genetic reagent (*D. melanogaster*)	*23E10-GAL4-AD;93F07-DBD*	Bloomington *Drosophila* Stock Center	RRID:BDSC-87032
Genetic reagent (*D. melanogaster*)	*23E10-GAL4-DBD*	Bloomington *Drosophila* Stock Center	RRID:BDSC-69269
Genetic reagent (*D. melanogaster*)	*30A08-p65-AD*	Bloomington *Drosophila* Stock Center	RRID:BDSC-71007
Genetic reagent (*D. melanogaster*)	*UAS-mCD8::GFP*	Bloomington *Drosophila* Stock Center	RRID:BDSC-5137
Genetic reagent (*D. melanogaster*)	*UAS-mCD8::GFP*	Bloomington *Drosophila* Stock Center	RRID:BDSC-5130
Genetic reagent (*D. melanogaster*)	*vGluT-Trojan-GAL80*	Bloomington *Drosophila* Stock Center	RRID:BDSC-60316
Genetic reagent (*D. melanogaster*)	*34F06-LexA*	Bloomington *Drosophila* Stock Center	RRID:BDSC-52759

Genetic reagent (*D. melanogaster*)	*34F06-GAL4*	Bloomington *Drosophila* Stock Center	RRID:BDSC-48125
Genetic reagent (*D. melanogaster*)	*UAS-NachBac*	Bloomington *Drosophila* Stock Center	RRID:BDSC-9466
Genetic reagent (*D. melanogaster*)	*LexAop-GAL80*	Bloomington *Drosophila* Stock Center	RRID:BDSC-32214
Genetic reagent (*D. melanogaster*)	*LexAop-GAL80*	Bloomington *Drosophila* Stock Center	RRID:BDSC-32213
Genetic reagent (*D. melanogaster*)	*UAS-GCaMP7s*	Bloomington *Drosophila* Stock Center	RRID:BDSC-80905
Genetic reagent (*D. melanogaster*)	*UAS-GCaMP7s*	Bloomington *Drosophila* Stock Center	RRID:BDSC-79032
Genetic reagent (*D. melanogaster*)	*20XUAS-IVS-phiC31*	Bloomington *Drosophila* Stock Center	RRID:BDSC-84155
Genetic reagent (*D. melanogaster*)	*20×UAS-SPARC2-l-mCD8::GFP*	Bloomington *Drosophila* Stock Center	RRID:BDSC-84144
Genetic reagent (*D. melanogaster*)	*Tsh-GAL80*	Gift from Wei Xie (Southeast University, Nanjing, China)	N/A
Genetic reagent (*D. melanogaster*)	*Otd-FLP,tubP>stop>GAL80*	Gift from Fang Guo (Zhejiang University, Zhejiang, China)	N/A

Genetic reagent (*D. melanogaster*)	*UAS-mGluR-RNAi*	Tsinghua Fly Center	THU5288
Genetic reagent (*D. melanogaster*)	*UAS-GluRIA-RNAi*	Tsinghua Fly Center	THU5238
Genetic reagent (*D. melanogaster*)	*UAS-GluRIB-RNAi*	Bloomington *Drosophila* Stock Center	RRID:BDSC-67843
Genetic reagent (*D. melanogaster*)	*UAS-Nmdar1-RNAi*	Tsinghua Fly Center	THU2118
Genetic reagent (*D. melanogaster*)	*UAS-Nmdar2-RNAi*	Tsinghua Fly Center	THU5862
Genetic reagent (*D. melanogaster*)	*UAS-GluRIIA-RNAi*	Tsinghua Fly Center	THU2659
Genetic reagent (*D. melanogaster*)	*UAS-GluRIIB-RNAi*	Tsinghua Fly Center	THU3089
Genetic reagent (*D. melanogaster*)	*UAS-GluRIIC-RNAi*	Tsinghua Fly Center	THU2049
Genetic reagent (*D. melanogaster*)	*UAS-GluRIID-RNAi*	Tsinghua Fly Center	THU2151
Genetic reagent (*D. melanogaster*)	*UAS-GluRIIE-RNAi*	Tsinghua Fly Center	THU3986
Genetic reagent (*D. melanogaster*)	*UAS-clumsy-RNAi*	Bloomington *Drosophila* Stock Center	RRID:BDSC-28351

Genetic reagent (*D. melanogaster*)	*UAS-KaiR1D-RNAi*	Tsinghua Fly Center	THU3982
Genetic reagent (*D. melanogaster*)	*UAS-Grik-RNAi*	Tsinghua Fly Center	THU3979
Genetic reagent (*D. melanogaster*)	*UAS-Ekar-RNAi*	Tsinghua Fly Center	THU3080
Genetic reagent (*D. melanogaster*)	*UAS-CG11155-RNAi*	Tsinghua Fly Center	THU3285
Genetic reagent (*D. melanogaster*)	*UAS-Kir2.1*	Bloomington *Drosophila* Stock Center	RRID:BDSC-6595
Genetic reagent (*D. melanogaster*)	*UAS-GFP1-10;LexAop-GFP11*	Gift from Chuan Zhou (Institute of Zoology, Beijing, China)	N/A
Genetic reagent (*D. melanogaster*)	*UAS-DenMark, UAS-syt::GFP*	Bloomington *Drosophila* Stock Center	RRID:BDSC_33065
Genetic reagent (*D. melanogaster*)	*CG7535-KO-LexA*	Gift from Yi Rao (Capital Medical University, Beijing, China)	N/A
Genetic reagent (*D. melanogaster*)	*UAS-Dicer*	Gift from Pengyu Gu (Sir Run Run Shaw Hospital, Zhejiang, China)	N/A
Genetic reagent (*D.*	*UAS-vGlut-RNAi*	Tsinghua Fly Center	THU2700

melanogaster)			
Genetic reagent (*D. melanogaster*)	*GluClα^{MI01156}*	Bloomington *Drosophila* Stock Center	RRID:BDSC-35096
Genetic reagent (*D. melanogaster*)	*GluClα^{glc1}*	Bloomington *Drosophila* Stock Center	RRID:BDSC-6353
Genetic reagent (*D. melanogaster*)	*Df(3R)BSC636*	Bloomington *Drosophila* Stock Center	RRID:BDSC-25726
Genetic reagent (*D. melanogaster*)	*84C10-GAL4*	Bloomington *Drosophila* Stock Center	RRID:BDSC-48378
Genetic reagent (*D. melanogaster*)	*78G01-GAL4*	Bloomington *Drosophila* Stock Center	RRID:BDSC-40009
Genetic reagent (*D. melanogaster*)	*per-GAL4*	Bloomington *Drosophila* Stock Center	RRID:BDSC-7127
Genetic reagent (*D. melanogaster*)	*Clk856-GAL4*	Bloomington *Drosophila* Stock Center	RRID:BDSC-93198
Genetic reagent (*D. melanogaster*)	*Clk4.1M-GAL4*	Bloomington *Drosophila* Stock Center	RRID:BDSC-36316
Genetic reagent (*D. melanogaster*)	*54D11-GAL4*	Bloomington *Drosophila* Stock Center	RRID:BDSC-41279
Genetic reagent (*D. melanogaster*)	*CNMa-GAL4*	Gift from Yi Rao (Capital Medical University, Beijing, China)	N/A

Genetic reagent (*D. melanogaster*)	*58H05-GAL4*	Bloomington *Drosophila* Stock Center	RRID:BDSC-39198
Genetic reagent (*D. melanogaster*)	*84A01-GAL4*	Bloomington *Drosophila* Stock Center	RRID:BDSC-48375
Genetic reagent (*D. melanogaster*)	*104y-GAL4*	Bloomington *Drosophila* Stock Center	RRID:BDSC-81014
Genetic reagent (*D. melanogaster*)	*Dh44-GAL4*	Bloomington *Drosophila* Stock Center	RRID:BDSC-51987
Genetic reagent (*D. melanogaster*)	*Lk-GAL4*	Gift from Yufeng Pan (Southeast University, Nanjing, China)	N/A
Genetic reagent (*D. melanogaster*)	*TH-D4-GAL4*	Gift from Mark N. Wu lab (Johns Hopkins University, Baltimore, UAS)	N/A
Genetic reagent (*D. melanogaster*)	*Hugin-GAL4*	Bloomington *Drosophila* Stock Center	RRID:BDSC-58769
Genetic reagent (*D. melanogaster*)	*Dlip2-GAL4*	Bloomington *Drosophila* Stock Center	RRID:BDSC-37516
